# Recurrent targets of aberrant somatic hypermutation in lymphoma

**DOI:** 10.18632/oncotarget.653

**Published:** 2012-11-12

**Authors:** Alireza Hadj Khodabakhshi, Ryan D. Morin, Anthony P. Fejes, Andrew J. Mungall, Karen L. Mungall, Madison Bolger-Munro, Nathalie A. Johnson, Joseph M. Connors, Randy D. Gascoyne, Marco A. Marra, Inanc Birol, Steven J. M. Jones

**Affiliations:** ^1^ Michael Smith Genome Sciences Centre, Vancouver, British Columbia, Canada; ^2^ Centre for Lymphoid Cancer, BC Cancer Agency, Vancouver, British Columbia, Canada; ^3^ Department of Pathology, University of British Columbia, Vancouver, British Columbia, Canada; ^4^ Department of Medical Genetics, University of British Columbia, Vancouver, British Columbia, Canada; ^5^ Department of Molecular Biology and Biochemistry, Simon Fraser University, Burnaby, British Columbia, Canada

**Keywords:** Aberrant somatic hypermutation, Genome wide study, Diffuse large B-cell lymphoma, Genomic rearrangements

## Abstract

Somatic hypermutation (SHM) in the variable region of immunoglobulin genes (IGV) naturally occurs in a narrow window of B cell development to provide high-affinity antibodies. However, SHM can also aberrantly target proto-oncogenes and cause genome instability. The role of aberrant SHM (aSHM) has been widely studied in various non-Hodgkin's lymphoma particularly in diffuse large B-cell lymphoma (DLBCL). Although, it has been speculated that aSHM targets a wide range of genome loci so far only twelve genes have been identified as targets of aSHM through the targeted sequencing of selected genes. A genome-wide study aiming at identifying a comprehensive set of aSHM targets recurrently occurring in DLBCL has not been previously undertaken. Here, we present a comprehensive assessment of the somatic hypermutated genes in DLBCL identified through an analysis of genomic and transcriptome data derived from 40 DLBCL patients. Our analysis verifies that there are indeed many genes that are recurrently affected by aSHM. In particular, we have identified 32 novel targets that show same or higher level of aSHM activity than genes previously reported. Amongst these novel targets, 22 genes showed a significant correlation between mRNA abundance and aSHM.

## INTRODUCTION

Physiological (normal) SHM occurs in immunoglobulin variable (IGV) loci (i.e. the portion of the gene encoding the variable region of immunoglobulin heavy chain) within germinal center (GC) B cells to generate antibody diversity. In normal GC B cells, SHM can also target the non-IGV loci such as the 5' sequences of the BCL6 and FAS/CD95 (TNFRSF6) genes [1, 2]. This process is initiated by cytosine deamination catalyzed by the activation induced (cytidine) deaminase enzyme (AID). The resulting uracils are then processed by the base excision repair or mismatch repair pathways. Faulty repair by these pathways in conjunction with replication via error-prone polymerases leads to a characteristic pattern of mutations that is a hallmark of somatic hypermutation events [3,4]. The mutation frequency in an IGV loci is estimated to be approximately 10^−3^ events per base pair which is 10^6^ fold higher than the spontaneous mutation rate in somatic cells [5]. The mutation frequency in a non-IGV locus is however, 50 to 100 times lower that of an IGV-locus [5]. SHM activity starts some 150 nucleotides downstream of the transcription start site (TSS) and extends typically a further two kilo bases into the gene [6]. However, the probability of mutation per base exponentially decreases with the increasing downstream distance to the TSS [7]. Due to the specific activity of AID acting on cytosines, the ratio of transition mutations over transversions is significantly higher than 1:2 that is expected on a random basis. Hot spot and cold spot patterns are also observed in the mutation pattern within a SHM-targeted region, indicating that SHM is influenced by the primary sequence of the DNA [8]. The most significant hotspot motif is the WRCY (where W denotes A or T; R denotes A or G; and Y denotes C or T) or its reverse complement RGYW [9]. There is also a strand-biased pattern in the targeted bases. Most notably, mutations at A:T base pairs are more likely to occur if A is located on the non-template strand of the gene. In addition, a C on the non- template strand can potentially induce a mutation in neighboring residues while a C on the template strand cannot [10]. Somatic hypermutation has been observed to aberrantly target the proto-oncogenes BCL6, PIM1, MYC, RHOH (RAS homologue gene-family member H) and PAX5 (paired box gene/protein 5); and the tumor suppressor gene CD95. Such mis-targeting of SHM contributes to the development of diffuse large B-cell lymphomas, tumors that derive from B cells within, or about to exit, the germinal center [8, 11–13], by providing a source of oncogenic mutations. More recently, through extensive sequencing of murine B-cell genes, it has been shown that selective targeting of AID and gene-specific, high-fidelity repair of AID-generated uracils are the two distinct mechanisms that protect genome from somatic hypermutation [14].

Aberrant SHM (aSHM) does not target proto-oncogenes in all subtypes of lymphomas originating from GC or post CG B-cells. In fact, aSHM activity in PIM1, PAX5, RHOH/TTF and MYC proto-oncogenes, have been acknowledged as a molecular feature exclusive to DLBCL. While aSHM of oncogenic loci affects more than 50% of DLBCL, it is rarely or never observed in other B-cell malignancies [12]. Somatic hypermutation has a driving role in chromosomal translocations in B-cell lymphomas [15]. These chromosomal aberrations usually cause dysregulation in the expression of oncogenes brought under the control of the IG loci. Somatic hypermutation intrinsically generates double-strand DNA breaks that are potentially recombinogenic [16]. A number of proto-oncogenes have been shown to be recurrent targets of aSHM in DLBCL (i.e. BCL6, MYC, RHOH/TTF, PIM1, PAX5 [2, 12], IRF4, ST6GAL1, BCL7A, CIITA, LRMP [17], BCL2 [18], and SOCS1 [19]). The first four genes identified through the targeted sequencing of only 17 selected genes in tumor samples [12]. This relatively high rate of positively identified genes among those analyzed suggested that somatic hypermutation is likely to target a wide range of genome loci. Although, in the past decade several studies have emerged to explain SHM mechanism and its role in tumorigenesis, to the best of our knowledge, there has not been an attempt to determine a comprehensive list of genome loci targeted at high frequency by aSHM. The aim of this study is to provide such a list in order to identify novel proto-oncogenes contributing to DLBCL.

## RESULTS AND DISCUSSION

We have performed a genome wide study on single nucleotide variations (SNVs) from whole genome data derived from 40 previously described DLBCL patients to identify recurrent SHM targets in DLBCL. Matched RNA seq and matched normal whole genome data was available for all of these samples and used in this study, however, only the SNVs derived from the whole genome data were included in the study as the variations derived from RNA-seq data are biased in the regions with high expression level. Our cohort consisted of 13 Activated B-cell (ABC) and 23 Germinal B-cell (GCB) subtypes (4 samples were not morphologically grouped).

Since SHM activities occur within a 2kb region downstream of TSS, we analyzed the mutations in this target region for annotated genes in the UCSC knownGene track [20]. We refer to these regions as SHMtargets throughout the text. Similar to previous studies in B-cell NHLs, we have determined criteria that reflect SHM activity in the target regions [8,11,12]. These measures include: (i) the pattern of mutations in the SHM-targets, (ii) the percentage of SNVs within a hot spot motif WRCY, (iii) the ratio of mutations at C:G sites to A:T sites and (iv) ratio of transition to transversion mutations. We defined an SHM indicator value for each SHM-target as the geometric mean of the p-values for measures (ii), (iii) and (iv). These are the measures that most commonly used to quantify SHM mutations. Supplementary Table 1 contains the calculated SHM measures for the SHM-targets with at least one SNV, sorted by the statistical significance of the observed mutations in the SHM-targets across the samples. Thus, the regions with high mutation rates are normally those observed to be recurrently mutated across multiple samples. We identified 44 potential SHM-targets, among over 46,000 analyzed regions, that were mutated at an equal or higher rate than those previously reported (See Table 1). The list includes all the 12 genes previously reported to be hypermutated in DLBCL (i.e. BCL2, BCL6, MYC, RHOH/TTF, PIM1, PAX5, IRF4, ST6GAL1, BCL7A, CIITA, LRMP and SOCS1). The signature of the SNVs in the SHM-target of these genes indicates the existence of the SHM in these region and in particular, 9 out of 12 genes show a significant associated SHM indicator value (i.e. less than 0.1). This supports the appropriateness of our analytical method. In addition to the previously reported genes, the list includes 32 novel recurrently mutated targets. These genes were identified as hypermutated on average in 8 (i.e. 20% of the samples) independent tumors and a median value of recurrence of 12 SNVs per SHM-target region. This list is enriched with genes that show indication of aSHM activity in their SHM-target regions. In particular, more than 81, 90 and 60 percent of the SHM-targets show a bias for SHM criteria (ii), (iii) and (iv), respectively. Furthermore, over 56% of these SHM-targets have an SHM indicator value less than 0.1. Table 1 shows somatic features for the recurrently mutated non-IG genes that are mutated at an equal or higher rate than previously reported SHM targets. There are however, genes with high mutation rates that lack the hallmarks of SHM activity. For instance, although mutated in 9 and 6 genomes respectively, the signature of SNVs associated with BACH2 and POU2AF1 are not indicative of any SHM activity. On the other hand, the key role of POU2AF1 in the formation of germinal centers [21] and the fact that BACH2 is involved in translocations in DLBCL [22,23] may indicate that the high mutation rate in these genes is associated with SHM or that mutation in these genes is under selection.

**Table 1:** Recurrent SHM-targets in DLBCL The list of the SHM-targets that are mutated at a rate equal or higher than known aSHM targets in B cells. The results are sorted by the number of mutations in the region (i.e. column 3). Columns 5, 6 and 7 are various feature values reported as the hallmark of SHM. These features were calculated after correction for base composition in the region (i.e. they are normalized by the frequency of the bases in those regions). The p-value associated for each feature is calculated using the exact Fisher test method. The last three columns are the transcript RPKM values corresponding to the target region that is extracted from RNA-seq data of the available samples.

Gene names	SHM indicator	Total SNVs	Mutated Samples	Transition/Transvertion (Pvalue)	Motif Bias (P-values)	C:G over A:T (P-value)	RPKM fold change between mutated vs. unmutated samples	Average RPKM in Tumor	Avearge RPKM Normal Bcell
BCL6*	0.1389	179	27	1.27(0.06)	1.41(0.0919)	0.77(0.5)	0.55739	61.4600	160.93086
BCL2*	0.2642	146	11	0.8(0.5)	1.47(0.0738)	0.79(0.5)	1.29298	20.7300	2.59639
**BTG2**	0.0123	55	18	1.04(0.45)	2.78(0.0002)	1.05(0.0172)	-0.27272	149.6800	223.5928
**TMSB4X**	0.0201	52	17	0.79(0.5)	1.69(0.1114)	1.41(0.0001)	0.11158	1485.8800	1017.2736
**ZFP36L1**	0.0000	52	16	1.17(0.29)	4.18(0)	1.26(0.0009)	0.05879	50.4900	142.76265
RHOH*	0.0509	42	17	0.68(0.5)	2.91(0.0005)	0.81(0.5)	0.01346	76.7300	352.06877
SERPINA9	0.1296	36	7	0.57(0.5)	2.15(0.0345)	1.03(0.1261)	5.48905	277.4700	237.10067
**CD83**	0.0006	34	8	1.13(0.37)	3.49(0.0001)	1.67(0)	1.08042	162.1900	478.47502
**SGK1**	0.0000	34	5	0.62(0.5)	5.5(0)	1.37(0.0103)	0.1586	2.9000	4.48411
BCL7A*	0.0083	32	14	1.46(0.14)	4.29(0)	0.9(0.5)	0.73039	31.1700	96.05465
BACH2	0.5000	30	8	0.25(0.5)	0.67(0.5)	0.75(0.5)	0.30362	8.0700	52.5643
**LTB**	0.0794	23	10	1.3(0.27)	2.72(0.0156)	1.15(0.1208)	1.81466	142.6400	189.28412
BIRC3	0.1158	21	12	1.1(0.41)	2.03(0.0975)	1.4(0.0385)	-0.10012	80.9500	175.95683
**HIST1H2AC**	0.0009	19	9	1.71(0.13)	4.95(0)	1.47(0.0123)	0	0.2000	0.08058
TCL1A	0.2012	17	8	0.55(0.5)	1.03(0.4869)	1.48(0.0335)	-0.07685	248.7300	709.73845
ST6GAL1*	0.2318	15	8	0.88(0.5)	2.17(0.1233)	1.03(0.202)	0.23782	64.4800	149.40245
**CD74**	0.0032	14	8	0.56(0.5)	5.18(0)	1.7(0.0061)	0.44198	10559.9000	8227.8865
SOCS1*	0.0272	14	5	1.33(0.3)	3.3(0.0117)	1.38(0.0058)	0.16955	26.1800	39.5316
IRF8	0.2448	13	9	1.6(0.2)	1.19(0.4275)	1.14(0.1694)	-0.0691	174.1000	462.84745
**BTG1**	0.0683	13	9	1.17(0.39)	3.55(0.0076)	1.22(0.1065)	0.12187	191.6600	975.71198
**CR607557**	0.0008	13	9	1.6(0.2)	6.69(0)	1.11(0.2004)	0	0.0000	0
LRMP*	0.2823	13	7	0.63(0.5)	1.08(0.4667)	1.48(0.0965)	0.22716	149.9900	276.99144
IRF4*	0.0208	13	4	5.5(0.01)	2.63(0.0714)	1.28(0.0201)	1.82701	106.0800	29.07161
CIITA*	0.0003	12	9	1(0.5)	6.29(0)	1.78(0.001)	0.49221	25.6600	23.75111
**DTX1**	0.0294	12	8	3(0.04)	3.71(0.0059)	1.26(0.1041)	0.42032	87.7300	151.20776
**CXCR4**	0.0025	12	7	0.71(0.5)	5.9(0)	1.68(0.002)	0.42432	143.9600	968.41417
PIM1*	0.0146	12	7	1(0.5)	4.6(0.0003)	1.47(0.0255)	0.96916	84.0200	165.35743
S1PR2	0.0183	11	7	1.75(0.18)	5.25(0.0005)	1.19(0.0689)	0.59678	22.3300	96.04705
MALAT1	0.1786	11	7	1.2(0.38)	2.6(0.0729)	1.21(0.2048)	0	0.0000	0
SPRED2	0.2356	11	6	0.57(0.5)	2.89(0.0523)	0.75(0.5)	1.46507	12.2400	22.09212
PAX5*	0.0114	10	7	1.5(0.26)	6.39(0.0001)	1.39(0.0726)	-0.2793	52.5200	127.01243
**DMD**	0.0239	10	3	2.33(0.1)	3.36(0.0301)	2.28(0.0044)	1.50279	10.5300	3.6875
LLT1	0.2591	10	3	2.33(0.1)	1.49(0.338)	0.49(0.5)	-0.21925	47.9800	86.73398
ETS1	0.1877	9	8	0.5(0.5)	2.08(0.2211)	1.61(0.0598)	0.40109	58.3700	102.81003
**DUSP2**	0.0040	9	4	2(0.16)	6.18(0)	1.18(0.0532)	0.65633	119.7600	160.9238
**AK123543**	0.0609	8	5	0(0.5)	4.1(0.0127)	1.71(0.0355)	0	0.0000	0
POU2AF1	0.5000	7	6	0.75(0.5)	0(0.5)	0.61(0.5)	-0.12034	153.9300	429.77219
GADD45B	0.1136	7	6	6(0.03)	2.58(0.1562)	0.93(0.3192)	-0.04866	30.9900	132.9862
MS4A1	0.1944	7	4	6(0.03)	0(0.5)	0.66(0.5)	0.03938	644.0700	715.41695
P2RY8	0.3182	7	3	1.33(0.35)	2.34(0.1826)	0.92(0.5)	0	0.4900	1.30263
GRHPR	0.1429	6	5	2(0.21)	0(0.5)	1.81(0.0282)	-0.17425	57.6200	27.42158
NCOA3	0.1770	6	4	5(0.05)	0(0.5)	1.39(0.2165)	0.22822	42.8100	76.49762
**UBE2J1**	0.0140	6	3	6(0.01)	5.29(0.0032)	1.57(0.1199)	-0.31589	67.8200	239.48779
MYC*	0.0630	6	3	1(0.5)	5.38(0.0029)	1.42(0.1713)	0.63538	22.5300	27.42303

Genes marked by a * / have been previously reported as targets of aSHM.

Genes with SHM indicator less than 0.1 are bold.

Since SHM activities are only associated with densely mutated regions, we would expect a decline in SHM feature values as the mutation rate decreases in the SHM-targets. In order to validate this hypothesis we divided the list of genes, sorted by mutation rate, into three groups. Expectedly, the IG loci were most highly ranked in the list. In particular, more than 20% of the top 60 SHM-targets belong to IG loci. Removing the IG loci and using them as a positive control group we divided the rest of the hypermutated SHM-targets into the following groups. Group I consists of the candidate SHM-targets (discussed earlier) that includes 44 SHM-targets with a mutation density above that of the known SHM targets. Group II consists of SHM-targets that are only moderately mutated. In particular, it includes SHM-targets that contain 3 to 5 SNVs observed in the input samples (92 SHM-targets) and group III consists of the remaining of the SHM-targets that contains at least two distinct SNVs (470 SHM-targets). Table 2 shows the average values of mutation features in each group as well as those in IG loci. As these data indicate the signals manifesting SHM activity degrades in SHM-targets with lower rate of somatic mutations. For instance, while the number of mutations in WCRY motif (after normalizing for base composition) is three times what is expected on a random basis in group I, it is only twice the random expected value in group II and is almost what is expected randomly in group III. Although some measures remain unchanged across groups, similar trends hold for many other measures as well, most notably, the SHM indicator measure that loses its significance by more than 2- fold in group II compared to group I.

**Table 2:** Average SHM feature values per group The average feature values in each group of SHM-targets. The last row contains the IG loci. Groups I, II and III are divided based on the mutation rate in the SHM-targets.

Groups	SHM indicator	Mutation enrichment in WRCY (P-value)	C:G over A:T (P-value)	Transition over Transversion (P-value)	Average RPKM in Mutated Samples	Average RPKM in Unmutated Samples	RPKM fold change	Average RPKM in Normal
Group 1 (mutation rate > 8e-5)	0.11	3.12(0.13)	1.25(0.17)	1.67(0.32)	502.7	357.1	0.59	463.3
Group 2 (mutation rate > 4e-5)	0.27	2.02(0.35)	1.25(0.33)	1.74(0.31)	50.96	57.34	0.03	74.4
Group 3	0.38	1.17(0.45)	1.1(0.51)	0.72(0.33)	50.29	50	0.03	48.72
IGH	0.14	2.7(0.15)	1.19(0.25)	1.3(0.31)	4482	2202	0.39	2846

**Figure 1:**
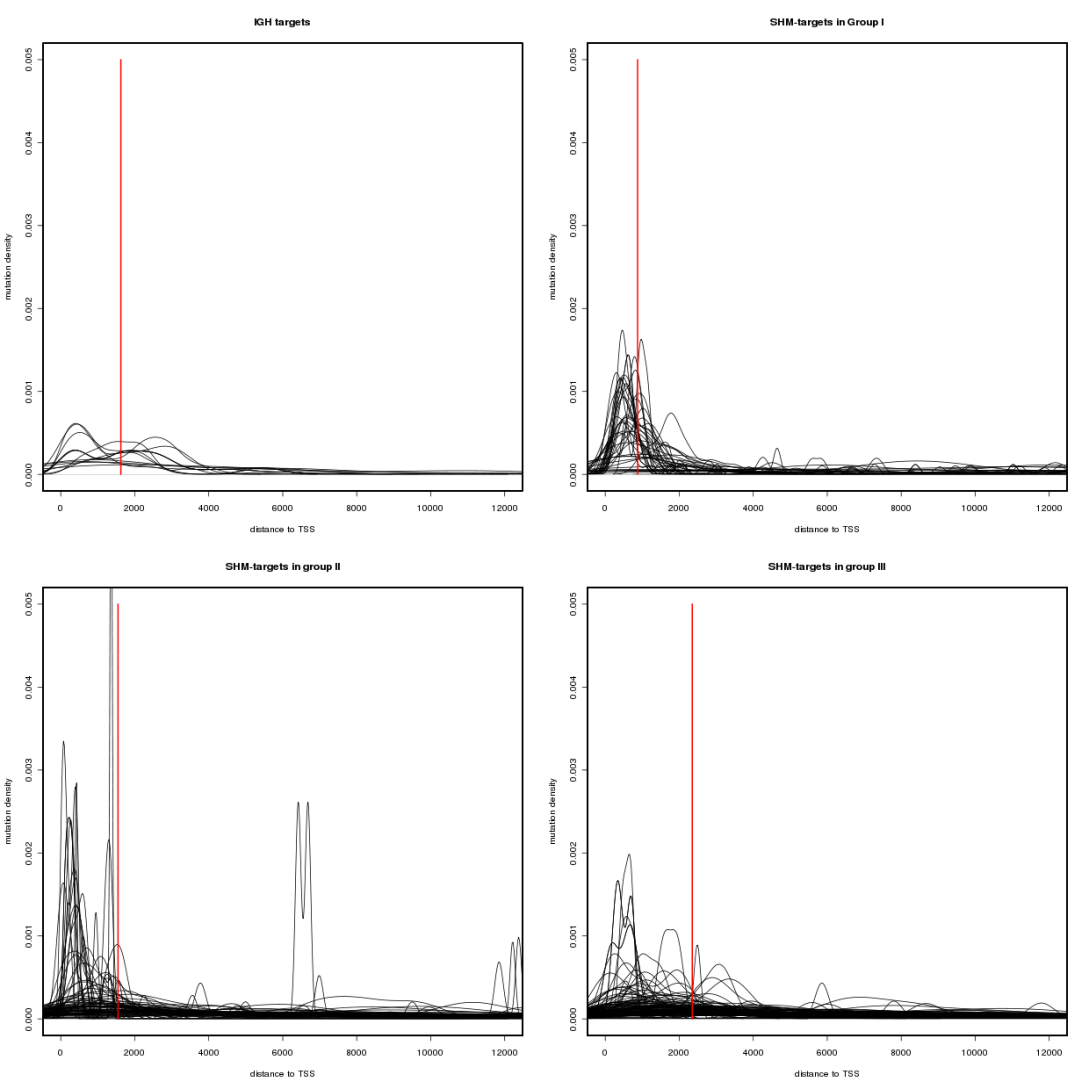
Mutation density in SHM-targets The mutation density curves in a 12 kb region downstream of transcription start sites. The red bars indicate the median of the SNV distance to the transcription start sites. As the plots show the concentration of SNVs moves further away from the transcription start sites as we move from group I to group III. A Two-sample Kolmogorov-Smirnov test (conducted using the ks.test R package) also suggests that the SNV distance distribution in group one is significantly different from that of group two and three (P < 2.2e −16) while the distance distributions in group two and three show a much higher degree of similarity (P = 0.03457).

Another indication of predominant SHM activity in the SHM-targets of group I and the IG group is the geographical pattern of mutations in these regions. Since the probability of mutations drops exponentially as the distance from TSS increases, the mutation density curve in the SHM-target region is expected to form a bell shape curve with its peak located in a region 150 to 1000 bases upstream the TSS. Such a trend can be observed more strongly in group I compare to groups II and III (see Figure 1). Figure 1 depicts the mutation density curve in a 12 kb region downstream of the TSS for the genes in each group. As the plots in this figure show the concentration of SNVs moves further away from the transcription start sites as we move from group I to group III. Furthermore, a two-sample Kolmogorov-Smirnov test (conducted using the ks.test R package) also suggests that the SNV distance distribution in group I is significantly different from that of group II and III (P < 2.2e −16), while the distance distributions in group II and III show a much higher degree of similarity (P = 0.03457).

**Figure 2:**
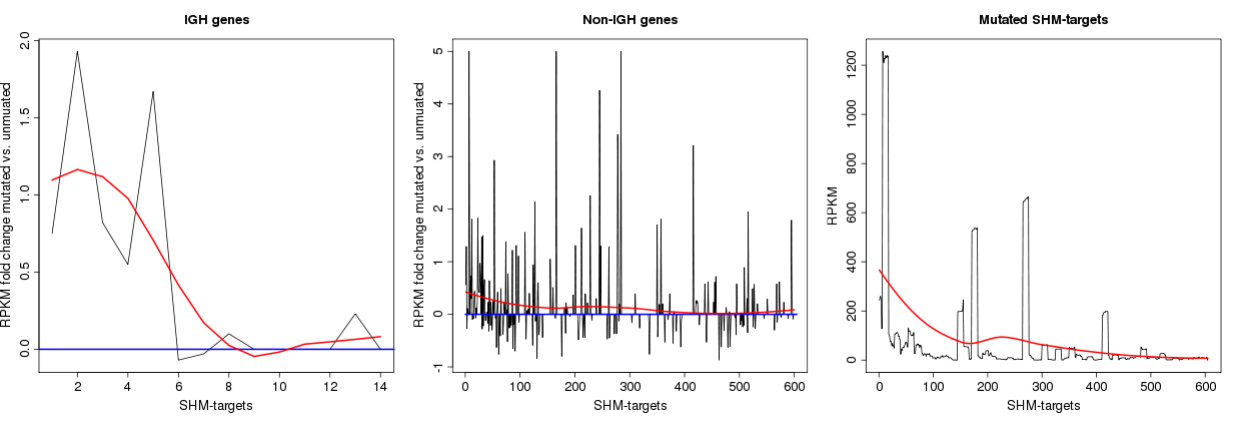
Transcription rate in SHM genes The left and middle plots depict RPKM fold change between mutated and unmuated samples in SHM-target region across IGH and non-IGH loci in group I, respectively. Here a positive value indicates an up-regulation in samples with mutation. Expression change is set to zero for the genes with low level of expression (i.e. RPKM less than 5). As the data in the middle plot suggests, there are more targets with a positive expression change amongst those with high mutation rate. More precisely, while over 70% of the target regions in group 1 are up-regulated in mutated samples, this ratio is 50% for targets in other groups (i.e. as expected on a random basis). The right plot depicts the average RPKM values for all the genes that has at least two mutations in their SHM-target region. The data in this plot shows that the absolute expression level in genes with higher SHM activities is also higher on average. The red smooth curves in the plots are polynomial regression fittings over the values computed using the loess R package. The targets on x-axis are sorted by mutation density in their SHM-target regions.

Another aspect of SHM is its reliance on active transcription. It has been shown that the elimination of transcription across an IG locus results in a loss of SHM [6] and also that the mutation rate of an IG gene is proportional to the level of transcription through that locus in a pre-B-cell line that supports SHM [24]. We investigated the correlation between transcription and the mutation in the SHM-targets using available RNA-seq data of the studied samples. These results show that in most of the cases the expression level of the targeted gene is higher in samples that are mutated compare to those that lack mutations in the SHM-target region (See Figure 2). In particular, 60% of the targets in group I show increase in RNA abundance by more than 10 percent (86% increase on average) in mutated samples while only in 16% of the cases the mutated samples have lower gene expression compare to samples with no mutation (14% decrease on average). The percentage difference is calculated as the difference between the RPKM values (mutated vs not mutated) divided by the sum of the RPKM values (See the last four columns in Table 2). This difference mostly reflects a trend towards higher mRNA abundance of the genes in the mutated samples, coinciding with the observation that gene expression promotes SHM. A statistical significance test also suggests that the expression of the genes that undergo SHM is significantly higher than the average expression of a randomly selected subset of the genes. More precisely, we generated multiple sets of k randomly chosen genes (where k is the number of genes in group I) from the genes with RPKM value over 1 (a total of 10800 genes), and performed a statistical significant test under the null hypothesis assumption that the average expression of the genes in group I comes from the same distribution governing the average expression of randomly selected set of genes. While a set of randomly selected genes has an average RPKM value of 50 the average RPKM value for the genes in group I is 350 resulting in a very significant p-value using a T-test (P < 10^−90^).

**Figure 3:**
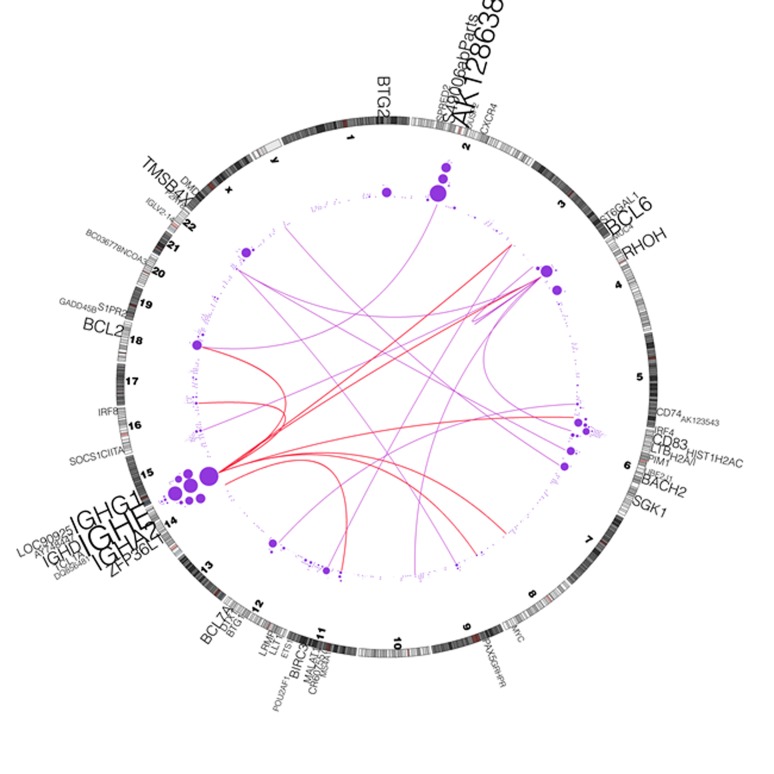
Correlation between mutations and rearrangements Distribution of somatic mutations in SHM-targets and correlation with genome rearrangements. A circos diagram [47] showing the distribution of somatic mutations in recurrently mutated SHM-targets and genomic rearrangements such as translocations and inversions. The purple circles represent the count of SNVs in the corresponding SHM-targets, and the arcs represent the chromosomal translocation events. The red and purple arcs represent translocation involving IGH loci and non-IGH loci, respectively. The size of the circles and the gene labels are proportional to the number of mutations in the SHM-target.

We also investigated the correlation between aSHM and translocations. To do so, we first identified genome wide translocation events independent from the results of the aSHM study. The translocation events were identified using ABySS [25], which assembles the short reads in the first stage and determines structural variations through alignment of the resulting sequence contigs. We used a curated subset of these candidate translocation events in our analysis. Figure 3, depicts the curated (arcs) chromosomal translocations in hypermutable genes along with the frequency of somatic SNVs within these genes. In particular, we found that 9 genes out of the 60 genes in group I (including IGH genes) are involved in validated translocations (i.e. 15% in the cohort). The 9 curated rearranged genes in our DLBCL samples are: BACH2, IGHD, BCL2, DQ856481, IGHE, PIM1, IGHA2, BCL6, abParts (2p11.2) and MYC. In addition, 9 additional genes in group I (i.e. CIITA, ZFP36L1, ST6GAL1, SGK1, IRF8, GRHPR, BIRC3, CD74 and AK128638) are found to be involved in transcloation in an independent DLBCL cohort based on the analysis of their available transcriptome data (the whole genome data were not available for this cohort to study the correlation with aSHM). In the screening of transcriptome data derived from 9 normal centroblast samples, none of these translocations were observed supporting the hypothesis that these events may be tumor specific. The correlation between translocation and SHM can be observed in Figure 3. In particular, synchronism can be seen between the translocation and mutation hotspots around 5q33 (CD74), 3q27 (BCL6), 18q21 (BCL2), 14q24 (ZFP36L1), 12q24 (BCL7A), 11q22 (BIRC3) and 16q24 (IRF8). The recurrence of genomic rearrangements and SHM has been reported previously for both BCL2 and BCL6 but not for any of the remaining genes [15, 26, 27]. Somatic hypermutation however, is not always present in the context of translocation although SHM occurs more frequently in the context of translocations in some genes such as BCL2 and BCL6. But even for these genes, SHM can target the region in the absent of translocation. In particular in nearly 30% of the cases with mutations affecting BCL2, no translocation event was observed in the vicinity of this gene (See Table 3). Other genes that are less commonly involved in translocations or other genomic rearrangements are also targets of SHM, but that our observation of SHM in the absence of rearrangements is consistent with the notion that hypermutation is occurring at these loci in the absence of (or possibly as a prerequisite to) the double-stranded breaks that result in these rearragements. Conversely, translocations may be selected for within a tumour by positioning a gene into a location where oncogenic SHM mutations are more likely to occur.

**Table 3:** Somatic hypermutation and genomic rearrangements Our observations show that somatic hypermutation commonly occurs in the absence of genomic rearrangements. Even for the BCL2 where aSHM previously reported in the context of (14:18) transclocation, we observed aSHM in the lack of any genomic rearrangment in several cases.

Gene	Samples with mutations and rearrangements	Samples with mutations only	Samples without mutations or rearrangement
BCL6	7	20	13
BCL2	8	3	29
BTG2	0	18	22
TMSL2	0	17	23
ZFP36L1	0	16	24
RHOH	0	17	23
SERPINA9	0	7	33
CD83	0	8	32
SGK1	0	5	35
BCL7A	0	14	26
BACH2	1	7	32
LTB	0	10	30
BIRC3	0	12	28
HIST1H2AC	0	9	31
TCL1A	0	8	32
ST6GAL1	0	8	32
CD74	0	8	32
SOCS1	0	5	35
IRF8	0	9	31
BTG1	0	9	31
LRMP	0	7	33
IRF4	0	4	36
CIITA	0	9	31
DTX1	0	8	32
CXCR4	0	7	33
PIM1	1	6	33
S1PR2	0	7	33
SPRED2	0	6	34
PAX5	0	7	33
DMD	0	3	37
CLEC2D	0	3	37
ETS1	0	8	32
DUSP2	0	4	36
POU2AF1	0	6	34
GADD45B	0	6	34
MS4A1	0	4	36
P2RY8	0	3	37
GRHPR	0	5	35
NCOA3	0	4	36
UBE2J1	0	3	37
MYC	1	2	37

We also screened recurrently targeted aSHM genes (i.e. the 44 genes in group I) against genes that are known to be cancer related. We selected a total of 3632 cancer related genes through a union of several credible cancer gene repositories including the CancerGenes database [28] that combines gene lists annotated by experts with information from key public databases and the Cancer Gene Census [29] that catalogues the genes for which mutations have been causally implicated in cancer. The abundance of cancer related genes in our recurrent aSHM targets shows that somatic hypermutation systematically targets genes that play a significant role in cancer development. More precisely, 29 genes (i.e. 66%) were found to exist in the cancer related genes (P < 10^−20^) out of which 13 genes (i.e. 30%) were known proto-oncogenes (P < 10^−4^).

The list of aSHM-targeted genes that have a role in cancer can be found in Supplementary Table 2. But perhaps more intriguing are the aSHM targets that have not been previously linked with cancer. These are are TMSB4X, SERPINA9, CD83, LTB, HIST1H2AC, CR607557, S1PR2, MALAT1, LLT1, AK123543, MS4A1 and UBE2J1. Abnormal regulation of some of these genes such as TMSB4X [30], SERPINA9 [31], CD83 [29] and LTB [32] has been observed in various types of cancer, including lymphoma. In addition, in the screening of the genes with reported variations in lymphoma from the COSMIC repository [33] and a collection of DLBCL related genes in the literature (See Supplementary Table 3) [12, 34–43], we confirmed that 21 of the genes in group I (See Supplementary Table 4), have been previously linked to lymphoma. While this finding on one hand shows that the recurrently aSHM targets are enriched with known lymphoma related genes (P < 10^−10^), on the other hand it shows that most of the mutations reported in this study are novel in lymphoma.

It would be of interest to determine whether differences exist in the mutations patters of individual genes in the Activated B-cell (ABC) and Germinal B-cell (GCB) subtypes of Group 1. We investigated this question on our cohort that consists 13 ABC and 23 GCB (4 samples have not been morphologically grouped). Our analysis shows that although some genes are favorably mutated in the samples of one subgroup (for instance BCL2, MALAT1, S1PR2 and SERPINA9 are mutated exclusively in GCB samples), however, the statistical power in our data is not sufficient to show that aSHM favorably targets one subgroup compare to another (See Supplementary Figure 1). For instance, although SERPINA9 is only mutated in GCB samples its P-value is 0.15 (Fisher's exact test) even before the multiple test correction.

The genes in group 1 were also tested for enrichment of certain functional classes using the DAVID functional annotation clustering tool. This revealed significant enrichment for genes involved in lymphocyte activation (P = 0.0056, Benjamini) and transcription factor activity (P = 0.0036, Benjamini) including known lymphoma-related genes such as the oncogenes BCL2 and BCL6 but also novel genes including CXCR4, RHOH, CD74 and MS4A1 (which encodes CD20, the target of the therapeutic monoclonal antibody rituxumab). The SHM targets were also enriched for genes involved in regulation of phosphorylation (P=0.008, Benjamini) including SOCS1, DUSP2, SGK1 and PIM1.

## CONCLUSIONS

We described genome wide recurrent targets of somatic hypermutations in diffuse large B-cell lymphoma. The mutation characteristics and distributions in the targeted regions resemble those of the SHM mutations in IGH loci and other known targets of aSHM in B-cell malignancies. Our analysis further confirms a significant concordance between genome rearrangements and SHM activities in the affected genes, an observation that has been previously reported. We observed aSHM at sites known ot be involved in translocations but in the absence of translocations, which is consistent with a model wherein aSHM may precede the genetic events that result in these rearrangements. It is possible that some of the observed SHM events arise in the B-cells prior to malignant transformation however unlikely, since it is known that other B-cell derived malignancies do not display aSHM events [12] other than those known to occur normally in B-cells [1, 2]. While the role of aberrant somatic hypermutation in malignant formation in lymphoma has been widely acknowledged, no effort has previously been made to comprehensively assess targeted genes. This work a comprehensive survey of genes affected by SHM. These data may help us to understand the mechanism by which SHM is targeted to proto-oncogene and provides a basis for DLBCL pathogenesis.

## MATERIALS AND METHODS

Sample preparation and sequencing were conducted as previously described [34]. The data is available in NCBI's Sequence Read Archive through accession code SRP001599. Variations were called using an in-house pipeline. Briefly, BWA [44] was used for alignment of sequence reads and then variants were called on genomic libraries using samtools-0.1.13 [45] pileup functionality, after the libraries have been merged and the duplicates marked using picard-tools-1.38 MarkDuplicates with default settings. An independent validation of the merge process compares the sum of the total number of reads of the individual lanes with that of the final files total number of reads to ensure data integrity. The resulting variant calls are filtered using samtools-01.13 varFilter and only those variants which pass the quality threshold of 20 were used in the analysis. We identified SNVs in the SHM-targets of the all the genes in the UCSC's knownGene track. Somatic putative SNVs were selected throughout filtering these tumor SNVs against the variations of the thousand genome projects and matched normals variants of the DLBCL patients. Each of these somatic putative SNVs were then validated using a bioinformatic approach through which the aligned reads in the tumor and the matched normal samples at the variation positions were analyzed case by case in order to eliminate germline variations or artifacts. More precisely, the variations that are also observed in the matched normal samples (i.e. germline variations) or those with low quality mapped reads were eliminated (at least 20% of the reads with mapping quality over 25 and base call quality over 10 are required to be mapped to the mutated allele in order to select a variation).

Here we explain how the statistical measures are calculated for the variations in SHM-target regions. Note that some of these measures are only included in the Supplementary tables and not in the main tables 1 and 2. The mutation density in each sample is calculated by diving the number of mutations in the SHM-target by the length of the SHM-target region. The average of mutation density across all samples is used as the mutation density for the corresponding SHM-target region. The p-value associated with each SHM-target is calculated using the Fisher's exact test by assigning the success rate as the probability that a somatic SNV occurs in the SHM-target region on a random basis and plugging in the number of somatic mutations across the genome and SHM-target region in the Fisher's exact test formula. These p-values are then corrected for multiple testing across all the SHM-target regions using the Benjamini method. The variation enrichment value in WRCY motif is the ratio of the number mutations in a WRCY motif over the expected number of mutations in a WRCY motif. The expected number of mutations in a WRCY motif is calculated by taking the percentage of the bases that occur in a WRCY motif in the SHM-target region and multiplying it to the number of SNVs in that SHM-target region. By assigning the percentage of the bases that occur within a motif as the success rate in Fisher's exact test, we calculated a significance value for the motif enrichment in each region. The enrichment and significance values for base specific SNVs (i.e. the enrichment of SNVs at G:C bases compare to A:T bases) are calculated in a similar manner. Note that these calculations take into account the base composition in the corresponding regions.

Translocation events were identified using ABySS [25] and then manual review was performed using IGV to view the reads to genome and exon-exon junction alignment of the RNA-seq data. Each library was viewed with 2 other libraries to establish whether the evidence for the rearrangement event looked credible.

Read alignments relative to the breakpoint and read mapping quality were taken into account. The gene expression values were calculated as reads per kilo base gene model per million mapped reads (RPKM) values from RNA-seq data derived from the tumors [46].

## Supplementary Figures and Tables




