# Supplementary material for: Recurrent targets of aberrant somatic hypermutation in lymphoma
Source: Oncotarget. 2012 Nov 12;3(11):1308–19. doi: 10.18632/oncotarget.653 (PMC3717795; doi:10.18632/oncotarget.653)
Supplement: Supplementary file 1 [file oncotarget-03-1308-s001.pdf]

# Recurrent targets of aberrant somatic hypermutation in lymphoma - Khodabakhshi et al

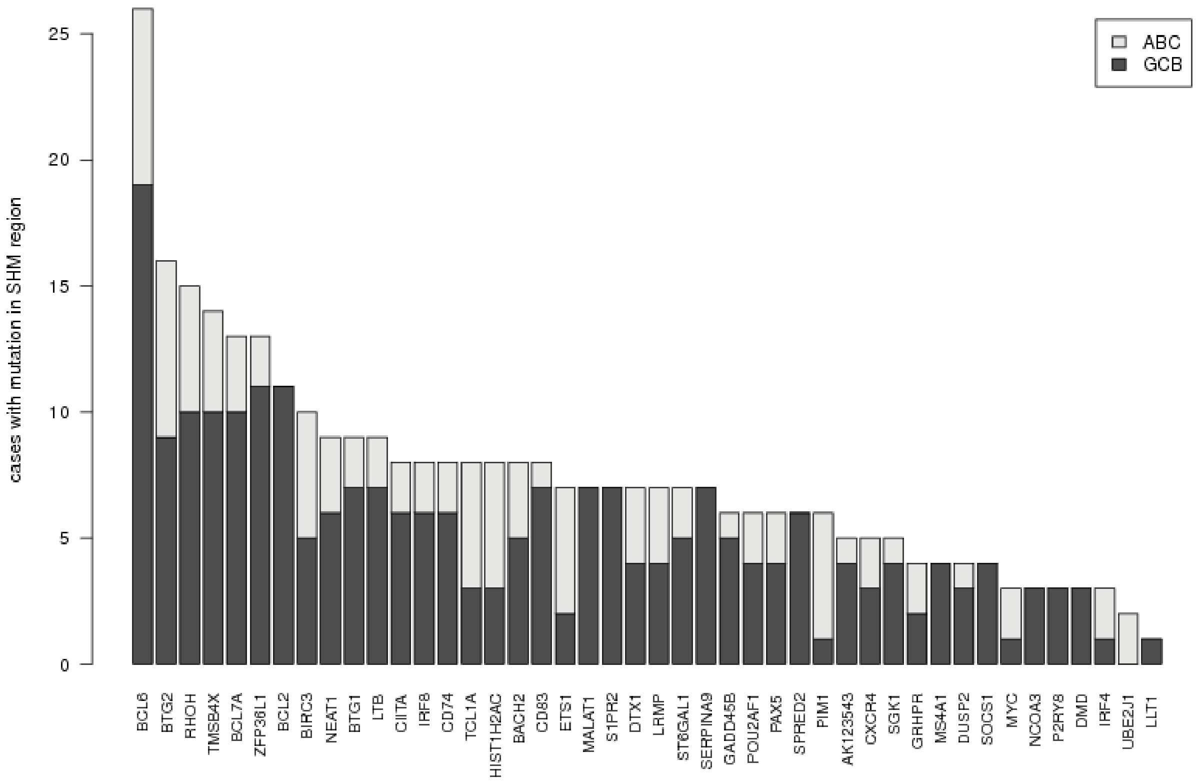


**Supplementary Figure 1: Mutation patterns in DLBCL subtypes.** The number of DLBCL cases with SHM affecting each of the genes in Group 1 for the two molecular subtypes. The total number of samples known to be ABC (n=13) and GCB (n=23) were compared for the number of cases with evidence for SHM in each of these genes. A sample was counted if it contained at least 1 somatic SNV in its SHM region. A trend towards SHM of certain genes in GCB cases was observed, for example, in BCL2, MALAT1 and S1PR2. A similar trend towards mutations in ABC cases was observed in other genes such as PIM1 and ETS1 but none of these trends met statistical significance due to limiting number of samples sequenced.
